# Degradable Biocompatible Porous Microtube Scaffold for Extended Donor Cell Survival and Activity

**DOI:** 10.1021/acsbiomaterials.2c00899

**Published:** 2023-01-03

**Authors:** Helen Nguyen, Chien-Chung Chen, Andreas Czosseck, Max M. Chen, Thomashire A. George, David J. Lundy

**Affiliations:** † Graduate Institute of Biomedical Materials & Tissue Engineering, College of Biomedical Engineering, Taipei Medical University, 250 Wuxing Street, Taipei 110, Taiwan; ‡ International Ph.D. Program in Biomedical Engineering, College of Biomedical Engineering, Taipei Medical University, 250 Wuxing Street, Taipei 110, Taiwan; § Medical Laboratory Science and Diagnostics, College of Medicine and Allied Health Sciences, University of Sierra Leone, Tower Hill, Freetown, Sierra Leone; ∥ Center for Cell Therapy, Taipei Medical University Hospital, 250 Wuxing Street, Taipei 110, Taiwan

**Keywords:** cell culture scaffold, fibroblast, host response, biomaterial implantation

## Abstract

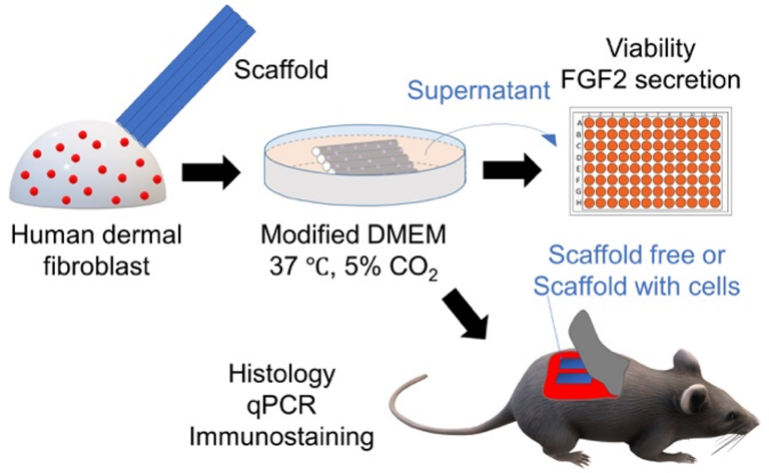

Cell therapy has significant therapeutic potential but is often limited by poor donor cell retention and viability at the host implantation site. Biomaterials can improve cell retention by providing cells with increased cell–cell and cell–matrix contacts and materials that allow three-dimensional cell culture to better recapitulate native cell morphology and function. In this study, we engineered a scaffold that allows for cell encapsulation and sustained three-dimensional cell culture. Since cell therapy is largely driven by paracrine secretions, the material was fabricated by electrospinning to have a large internal surface area, micrometer-thin walls, and nanoscale surface pores to allow for nutrient exchange without early cell permeation. The material is degradable, which allows for less invasive removal of the implant. Here, a biodegradable poly(lactic-*co*-glycolic acid) (PLGA) microtube array membrane was fabricated. *In vitro* testing showed that the material supported the culture of human dermal fibroblasts for at least 21 days, with paracrine secretion of pro-angiogenic FGF2. *In vivo* xenotransplantation of human cells in an immunocompetent mouse showed that donor cells could be maintained for more than one month and the material showed no obvious toxicity. Analysis of gene expression and tissue histology surrounding the implant showed that the material produced muted inflammatory and immune responses compared to a permanent implant and increased markers of angiogenesis.

## Introduction

*In vitro* cell culture is a powerful tool used in many biomedical research fields. Cells may be used for drug activity screening and toxicity testing, for understanding molecular pathways and their perturbations, or as factories for the production of proteins or exosomes. Many cells have also been explored as therapeutics, where they are implanted into the body to replace or supplement host functions.

Cell culture typically relies on a monolayer of cells attaching to rigid, two-dimensional (2D) plastic surfaces. The dishes and flasks that are routinely used in most laboratories are formed from plasma-treated polystyrene.^1^ Polystyrene has many practical advantages, including transparency, low cost, and high stability. However, adherent cell culture on such 2D surfaces forces cells to adopt an unnatural, flat, polarized morphology with attachments present on the basal surface only. As a result, cells form few apical surface attachments and have reduced cell–cell interactions and cell–matrix interactions.^2,3^ It is well-known that, in both native tissues and *in vitro* systems, the physicochemical properties of the extracellular matrix affect cell morphology, survival, proliferation, gene/protein expression, and response to stimuli.^4−6^ In addition, gradients of nutrients, oxygen, and soluble factors alter cell behavior, all of which are disrupted during 2D cell culture. For example, it has been previously demonstrated that cancer cells display notably different drug susceptibility in 2D and 3D, which can be explained by altered expression of drug targets, greater cell resilience, or simply by the heterologous diffusion of drugs in the tumor microenvironment.^7^ Owing to the greater predictive power of 3D cell culture, some drug companies are integrating 3D cell culture technologies into their drug discovery processes.^8^ Recently, new technologies enabling culture of cell spheroids and organoids in high throughput-compatible formats have been described.^9,10^ However, for routine culture of large numbers of cells in most research laboratories, 2D cell culture remains the standard.

Cell therapy is based on the implantation of donor cells for therapeutic purposes. These cells may be obtained from HLA-matched allogeneic donors, or autologous cells may be isolated from a patient, expanded, or processed *ex vivo* and then reintroduced to a target site.^11^ Donor cells may integrate into the host tissue and replace their function or they may act *via* the secretion of trophic factors such as growth factors, cytokines, miRNAs, and exosomes.^12,13^ However, cell therapy is limited by low retention and survival of cells at the implantation site.^14^ During the preparation of a cell suspension, enzymes are used to digest cell–cell and cell–matrix attachments and then cells are subjected to centrifugation forces, temperature changes, shear forces, and the hostile microenvironment of the injured host tissue. As a result, many injected cells rapidly die due to anoikis or are lost from the target tissue, thus limiting overall therapeutic usefulness.^15^ Combining cells with implantable biomaterials offers some solutions to these problems, allowing cells to maintain cell–cell/cell–matrix contacts and avoiding many stresses associated with cell preparation and injection.^16,17^ Biomaterials can also retain donor cells at the target site and offer protection from cellular components of the host immune system, depending on their porosity. In some applications, long-term placement of cells may be desired, such as encapsulated pancreatic islet cells for blood glucose control, which is a lifelong treatment. However, in other applications such as acute wound healing, only temporary survival of the cells is necessary. In these instances, a degradable material would be advantageous, avoiding the need for surgical removal.

Following implantation, foreign materials are rapidly covered with adsorbed proteins and host cells are recruited to the area. Circulating neutrophils, monocytes, and tissue macrophages adhere to the foreign material, and surrounding tissue fibroblasts differentiate into collagen-secreting myofibroblasts.^18,19^ The intensity and duration of the host response varies depending on many factors including the location of the implant site, the material surface properties, material dimensions, and attributes of the host.^19^ Implants that cannot be degraded and removed may be subsequently surrounded by a capsule of fibrous material, which can interfere with implant function, cause patient discomfort, and produce undesired aesthetic outcomes.^20,21^ For supporting cell therapy, a degradable material would allow for noninvasive removal of the implant, which would be advantageous for some applications where a permanent implant is not required. Therefore, we sought to develop and fabricate a degradable biomaterial, which would be suitable for delivering and retaining donor cells at an injury site.

Three-dimensional scaffolds for cell culture and therapy have been formed by many methods including internal phase emulsion, decellularization of native tissues, bioprinting, gas foaming, porogen leaching, and others.^22,23^ Electrospinning of polymers is also widely used since it offers a high degree of control and creation of scaffolds with structures similar to native tissue ECM. Hollow fibers or microsized capillary tubes are an attractive method for 3D cell culture. Cell suspensions can be taken up into the microtubes by capillary action, and the structure provides a large surface area to volume ratio. In this study, we utilized a coaxial electrospinning technique to create a series of interlinked hollow tubes, which we adapted to form a scaffold suitable for cell culture.^24^ Here, we describe a methodology for the fabrication of a scaffold formed from poly(lactic-*co*-glycolic acid) (PLGA). PLGA is currently used in many products approved by the United States Food and Drug Administration including drug delivery depots, degradable sutures, and grafts.^25^ After implantation, PLGA degrades rapidly by hydrolysis reactions, forming lactic acid and glycolic acid, which are easily metabolized and removed by tissues. As a result, PLGA tends to provoke mild, short duration host responses following implantation.^26^ Thus, we hypothesized that PLGA-based scaffolds would have good biocompatibility and allow for short- to medium-term donor cell retention at the implantation site. As a comparison, we formed similar scaffolds using polysulfone (PSF), a highly stable thermoplastic polymer that is not degradable in the body.^27^ PSF is also used clinically but is restricted to fewer applications such as corneal implants.^28^ We expected that the PSF-based materials would allow for a longer duration of donor cell retention and survival than PLGA. To test these hypotheses, scaffolds were formed from electrospun PSF and PLGA and implanted into mice. Empty materials were used to examine biocompatibility, and materials encapsulating human cells in a mouse xenotransplantation model were used to determine cell retention.

## Materials and Methods

### 2.1 Material Preparation

#### 2.1.1 PSF and PLGA Microtube Array Membrane Scaffold Formation

Both materials were formed using a coaxial (core–shell) electrospinning method.^24^ PSF shell solution was prepared by dissolving polysulfone (PSF, 35 kDa, Sigma-Aldrich) and polyvinylpyrrolidone (PVP, 1.3 MDa, Sigma-Aldrich) in a mixture of tetrahydrofuran (THF, JT Baker) and dimethylacetamide (DMAC, Alfa Aesar) to obtain a concentration of 16% (w/v), in which the PSF/PVP ratio was 75:25. Core solution was prepared by dissolving polyethylene glycol (PEG, 35 kDa, Sigma-Aldrich) and poly(ethylene oxide) (PEO, 900 kDa, Sigma-Aldrich) in distilled water to obtain a concentration of 8–12% (w/v), in which the PEG/PEO ratio was 50:50. The two solutions were simultaneously electrospun, applying a voltage of 6.0 ± 0.5 kV to the system. The electrospinning process was conducted in a clean room at 23 ± 2 °C and 60 ± 5% *H*. PLGA shell solution was prepared by dissolving poly(lactic-*co*-glycolic acid) (PLGA 75:25) and polyethylene glycol (PEG, 35 kDa, Sigma-Aldrich) in dichloromethane to obtain a concentration of 26.5% (w/v), in which the ratio PLGA/PEG was 80:20. Core solution was prepared by dissolving polyethylene glycol (PEG, 35 kDa, Sigma-Aldrich) and poly(ethylene oxide) (PEO, 900 kDa, Sigma-Aldrich) in distilled water to obtain a concentration of 12–14% (w/v), in which PEG/PEO ratio was 50:50. The two aforementioned solutions were simultaneously electrospun by applying a voltage of 5.75 ± 0.75 kV to the system. The electrospinning process was conducted in a clean room at 23 ± 2 °C and 60 ± 2% *H*. The collected raw scaffolds were soaked in distilled water for 24 h to remove porogen, dried under sterile conditions, and then cut to the desired size.

#### 2.1.2 Gold Coating and SEM

Samples were mounted onto a holder, gold-coated for 60 s, and then observed by a Hitachi TM3030 scanning electron microscope (SEM) at an accelerating voltage of 15 kV. SEM images were analyzed in ImageJ. At least *n* ≥ 20 tube diameters and heights were measured for each scaffold. The inner wall thickness and outer wall thickness were measured at *n* ≥ 20 sites. The surface pore diameter was measured for *n* ≥ 200 pores. To analyze samples seeded with cells, early time points (3 days) were used to avoid overconfluence of cells. Samples were fixed, dehydrated, and critical point dried, and the upper surface of the scaffold was removed by tape stripping to expose the inside of the tubes. Samples were then gold coated and imaged.

#### 2.1.3 Mechanical Testing

Tensile strength was assessed using a Cometech QC-528M1. Elongation speed was set at 6 mm per minute, and the measurement was conducted with 0.05 kgf preload on the specimens to remove slack. The linear load–displacement data set was then converted into a stress–strain curve.

#### 2.1.4 Plasma Treatment

Materials were plasma treated with oxygen using a basic plasma treater (Harrick, PDC-32G) at low (7W), medium (10W), or high (18W) power for 5, 10, or 20 min. The degree of hydrophobicity was then measured by goniometry (*N* = 4 samples per condition).

#### 2.1.5 Contact Angle Measurement

Water contact angle was measured using a GBX DigiDrop contact angle meter. Materials were mounted on a glass slide, which was slowly moved upward to contact a suspended water droplet. The contact angle was measured at the water, material, and air intersection five seconds following water contact. There were four replicates for each condition tested. Parafilm was used as a hydrophobic control.

#### 2.1.6 Fourier-Transform Infrared Spectroscopy (FTIR)

FTIR was performed using a Nicolet iS10 FTIR machine linked to a MicromATR vision accessory. Each sample (*n* = 3 batches per material) was recorded 50 times from 4000 to 400 cm^–1^ at ambient temperature. A background scan was performed before recording the sample spectrum, and the apparatus was wiped with ethanol 75% between runs.

#### 2.1.7 Thermal Analysis

Thermogravimetric (TGA) measurement of each material was performed using a Hitachi STA7300. The weighed sample was heated with nitrogen gas at a rate of 10 °C per minute with a gas flow rate of 100 mL per minute. PLGA materials were measured between 20 and 500 °C, and PSF materials were measured from 20 °C to 1000 °C.

#### 2.1.8 Mass Loss Calculation

Freshly made, dry samples from multiple batches were weighed and then incubated in 5 mL sterile PBS, pH 7.4, at 37 °C. Since a single scaffold weighs only 1–4 mg, 10 scaffolds were pooled to form one “sample” to allow for accurate weighing. At each time point (1, 2, 4, and 8 weeks), four samples were removed, dried, and weighed. Mass loss was then calculated based on the original sample weight.

### 2.2 Cell Culture

#### 2.2.1 Cell Culture

Human dermal fibroblasts (HDFs) from a healthy donor were purchased from the Bioresource Collection and Resource Centre, Taiwan (CG1631). HDFs were cultured in Dulbecco’s modified Eagles medium with low sodium bicarbonate, 4 mM l-glutamine, 4500 mg/L glucose, and 1 mM sodium pyruvate (ATCC, 30-2002), supplemented with 15% (v/v) FBS (Hyclone). Cells were cultured at 37 °C with 5% CO_2_. To load cells inside the hollow tubes of each material, an 8 μL cell suspension was placed onto a sterile surface and the open tube end of the scaffold was placed into the suspension. Cells were then taken up by capillary action. Loading of each scaffold took 1–3 min depending on the polymer used, the tube diameter, or the degree of plasma treatment. Loading was verified by light microscopy, and cultures with excessive cell attachment to the outside of the material were discarded. Tube ends were sealed by gently pinching with tweezers.

#### 2.2.2 Viability Assays

Cell metabolic activity was measured using a nontoxic CCK-8 (Boster) assay. Culture medium containing 10% (v/v) reagent was added to a fresh well, and the scaffold cultures were incubated in the well for the reaction to take place. The material was then removed, and the supernatant absorbance was read at 450 nm by a spectrometer (*n* ≥ 5 samples per time point). An empty scaffold containing no cells was used as a blank for each run. To stain protein, cell-loaded or empty cultured materials were stained with Coomassie dye at room temperature and then destained with multiple changes of 50% ethanol until the materials without cells were destained.

#### 2.2.3 Cell Culture Supernatants Were Harvested after 3 Days of Media Conditioning

The supernatant was centrifuged (2000*g*, 10 min) to remove debris; then, human FGF2 protein was quantified by using an ELISA kit (Abcam, ab246531) following the manufacturer’s protocol. Nonconditioned culture medium was used as a blank. *N* = 5 for 2D culture, *N* = 8 for encapsulated culture.

### 2.3 Animal Experiments

#### 2.3.1 Animal Surgery

Experiments were carried out with ethical approval under protocol numbers LAC-2019-0207 and LAC-2021-0225, Taipei Medical University. C57/BL6 mice (9 weeks old, male) were purchased from Lasco, Taiwan and housed at Taipei Medical University Laboratory Animal Housing Centre with a 12/12 light/dark cycle and *ad libitum* access to food and water. After 1 week of acclimatization, mice were anesthetized with inhaled isofluorane, hair was removed by shaving and depilatory cream, and the surgical site was swabbed with povidone iodine. To form an ischemic flap model, a 10 (W) × 20 (L) mm^2^ incision was created along the midline to form a skin flap and the subcutaneous space was fully separated from underlying tissue by dissection using microscissors.^29^ PSF or PLGA-based materials were either empty or seeded with 8 × 10^4^ live human dermal fibroblasts (PSF- HDF/PLGA-HDF), which were labeled with a long-lasting fluorescent dye (CellTracker, Thermo, C34552). Scaffold corners were trimmed to avoid inducing irritation to the animals, resulting in a material 2.0 × 0.5 cm^2^. Two scaffolds were implanted per animal: one on the left and one on the right. The skin was then closed with 6/0 silk, with eight stitches on each longitudinal edge and five along the caudal edge. A sham surgery was also carried out, replicating the same skin incisions, tissue dissection, and closing sutures but without material implantation. In the PSF material group, one mouse in the D43 group removed sutures from the skin flap and was excluded from analysis. All other groups contained three animals. Mice were sacrificed after 7 days and 43 days. Blood was collected by cardiac puncture into pediatric EDTA or SST tubes (BD Biosciences) and analyzed, with complete blood count (CBC, Idexx ProCyte) and biochemical analyses (Idexx VetTest).

#### 2.3.2 Determination of Cell Retention

Cell retention in *ex vivo* tissues was measured by a Lumina III XMRS *In Vivo* Imaging System at excitation 580 nm and emission 620 nm. Positive and negative controls were performed using labeled cell-loaded materials and empty materials. The sham group was used as a negative control for the background signal, included in every frame. Fluorescence signal (radiant efficiency) was determined by selection of an ROI of the sample using the provided LivingImage software. All images are presented at the same acquisition settings and color scale. Retention% was calculated by comparing the *ex vivo* samples to *in vitro*-cultured samples, which served as a 100% retention control. Samples from all animals were analyzed.

#### 2.3.3 Gene Expression Analysis

Tissues surrounding the implanted material were washed in PBS, then placed into TRIzol reagent (Thermo) and immediately snap frozen in liquid nitrogen, and then stored at −80 °C until analysis. Samples were homogenized (MagnaLyser, Roche), and RNA was extracted using spin columns (Qiagen, 74004) and quantified by a microplate reader. Reverse transcription was performed using SuperScript IV (Invitrogen, 18-090-010) in a Thermo StepOne thermocycler following the manufacturer protocol. Specific primers (listed in Table S1) were added (200 nM) used to examine expression of mouse genes associated with tissue remodeling, immune cells, macrophages, and angiogenesis using SYBR green (Thermo, 43-687-08) and an ABI 7500 PCR system. Each primer was also tested without a cDNA template and was confirmed to lack amplification. *Gapdh* was used as an internal control for each sample (typical cycle threshold (CT) of 18–20), and gene expression was expressed relative to sham-operated animals by the ΔΔ CT method. A heatmap was generated in GraphPad Prism 9.

#### 2.3.4 Histology and Imaging

Tissue sections from all animals were fixed with 4% paraformaldehyde overnight, dehydrated through graded ethanols, then paraffin embedded. Four μm sections were cut then stained with anti-aSMA-Cy3 (Sigma, C6198), anti-F4/80-AF647 (BioLegend, 123122), anti-CD3-BV421 (BioLegend, 17A2) and phalloidin-iFluor488 (AbCam, ab176753) to visualize myofibroblasts, macrophages, T-cells and F-actin, respectively. Images were acquired using a Zeiss Stellaris confocal imaging system and Zeiss LAS X software. Additional sections were stained with Masson’s Trichrome following standard laboratory methods. Two sections per animal were examined, and representative images are shown.

### 2.4 Data Handling and Statistical Analysis

Data were collected and preliminary calculations, such as subtraction of blanks, were made in Microsoft Excel or Google Sheets. GraphPad Prism 9.3.1 (Mac) was used to generate graphs and perform statistical analyses. The sample sizes and statistical tests used are described in the relevant figure legends and individual data points are shown on graphs where possible. Data in graphs show the mean ± standard error, unless otherwise stated. Multiple histology images were stitched using Affinity Photo 1.10.5 (Mac). If focus stacking was necessary, this was also performed using Affinity Photo. Final figures were assembled in Microsoft Powerpoint.

## Results

### 3.1 Material Fabrication

#### 3.1.1 Material Fabrication

PSF and PLGA microtube membrane array scaffolds were electrospun, washed to remove solvents and porogens, and then inspected by macroscopic and scanning electron microscopy (SEM) imaging.

**Figure 1:**
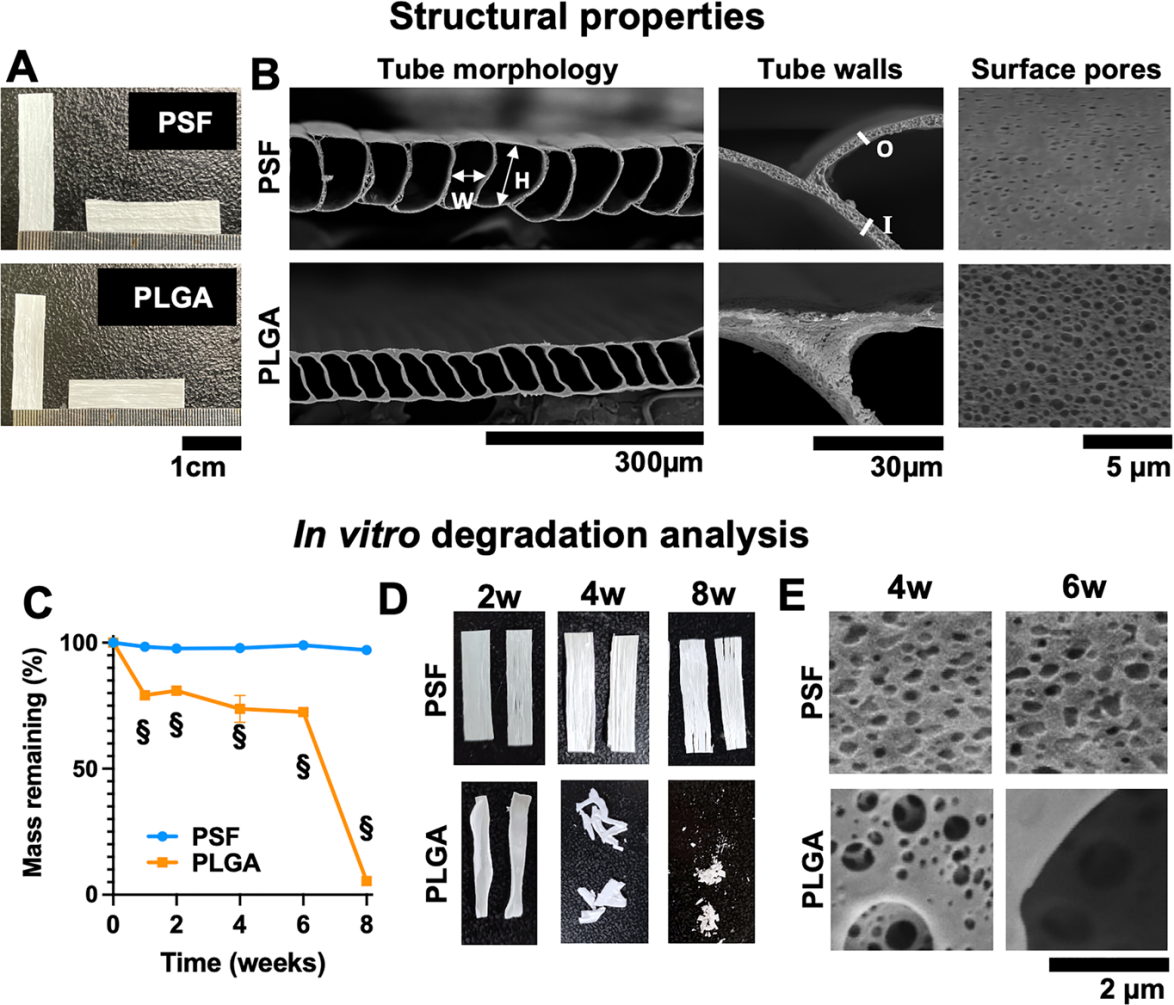
(A) Macroscopic images of PSF and PLGA-based microtube membrane scaffolds. (B) Representative SEM images showing scaffold structure. Adjoined hollow tube consistency, internal/external wall thickness, and surface nanoscale pores are highlighted. W, tube width; H, tube height; O, outside wall; I, inside wall. (C) Degradation measured by mass loss% under *in vitro* conditions. *N* = 4 samples of 10 scaffolds per data point. § denotes *p* ≤ 0.001, determined by unpaired *t*-test. (D) Representative macroscopic images of material degradation under controlled conditions. (E) Representative SEM images of surface pores at 4 and 6 weeks.

Representative macroscopic images of the final materials are shown in Figure 1A. Both materials formed thin, flat, slightly transparent sheets that could be cut to 2.0 × 0.5 cm^2^ rectangles. After optimization, both materials could successfully be electrospun to produce hollow tubes/capillaries with thin walls and large surface pores, as shown in Figure 1B. Quantification of tube dimensions (Table 1) showed that PLGA-based materials still had tube widths and heights smaller than PSF-based scaffolds. In terms of pore diameters, essential for nutrient exchange, PSF and PLGA both formed similar-sized surface pores of 495 ± 101 and 595 ± 208 nm, respectively. However, the pore density of PLGA scaffolds was almost 6-fold higher than that of PSF. Pores of up to 800 nm have been previously shown to allow for the secretion of donor cell products while reducing host cell infiltration.^30^

**Table 1:** Structural Properties of PSF and PLGA-Based Scaffolds^a^

	PSF	PLGA	*P* value
tube height (μm)	124.6 ± 15.6	90.7 ± 5.3	0.002
tube width (μm)	68.8 ± 8.4	32.6 ± 4.9	<0.0001
inner wall (μm)	2.6 ± 0.5	3.6 ± 1.3	ns
outer wall (μm)	3.8 ± 1.1	5.0 ± 2.0	ns
surface pores (nm)	495 ± 101	595 ± 208	ns
pores per 100 μm^2^	44 ± 4	262 ± 11	<0.0001

a Numbers show mean ± SD. *P* shows statistical significance determined by unpaired *t*-test (*n* = 5 batches).

These parameters provide approximately 5.42 cm^2^ of surface area and 12.01 μL of internal volume for PSF scaffolds. PLGA scaffolds, owing to smaller tube dimensions, had a lower internal volume of approximately 8.17 μL. However, due to a greater number of individual tubes, the surface area available for growth was larger than PSF, calculated at approximately 6.81 cm^2^.

Since our goal was to fabricate a biocompatible, biodegradable scaffold, the timeline of mass loss of each final product was measured under controlled conditions, as shown in Figure 1C. The mean starting weights for an individual scaffold were PSF = 1.31 ± 0.08 mg and PLGA = 3.51 ± 0.24 mg. After 7 days, PSF materials lost 1.59 ± 0.05% of their mass, whereas PLGA lost 20.8 ± 3.3% (*p* = < 0.0001), showing that, as expected, PLGA degraded much more rapidly than PSF. Between 2 and 4 weeks, the PLGA broke into smaller fragments, as shown in Figure 1D. After 8 weeks, very little PLGA mass (5.45 ± 2.04%) remained. Visual analysis of the material surface by SEM (Figure 1E) revealed that PSF did not noticeably change but PLGA-based scaffolds showed openings on the surface of up to 1 μm after 4 weeks and large cavities of up to 5 μm diameter after 6 weeks. At 8 weeks, PLGA scaffolds had degraded into a fine powder. Taken together, these results show successful electrospinning of microtube membrane array scaffolds from PLGA, which were degradable in approximately two months under controlled *in vitro* conditions. Degradation *in vivo* would be expected to occur more rapidly due to physical disruption, enzymatic activity, and the host immune response. Since PLGA may shrink after immersion in solutions, we measured the tube diameters before and after 7 days incubation in PBS, pH 7.4 at 37 °C, as shown in Figure S1A. The results show a statistically significant reduction in tube height from 90.7 to 85.9 μm. Tube width reduced from 32.6 to 30.0 μm but this was not statistically significant (*p* = 0.063). Taken together, it is unlikely that this change would meaningfully alter the application of scaffold for cell culture or implantation.

#### 3.1.2 Material Characterization

**Figure 2:**
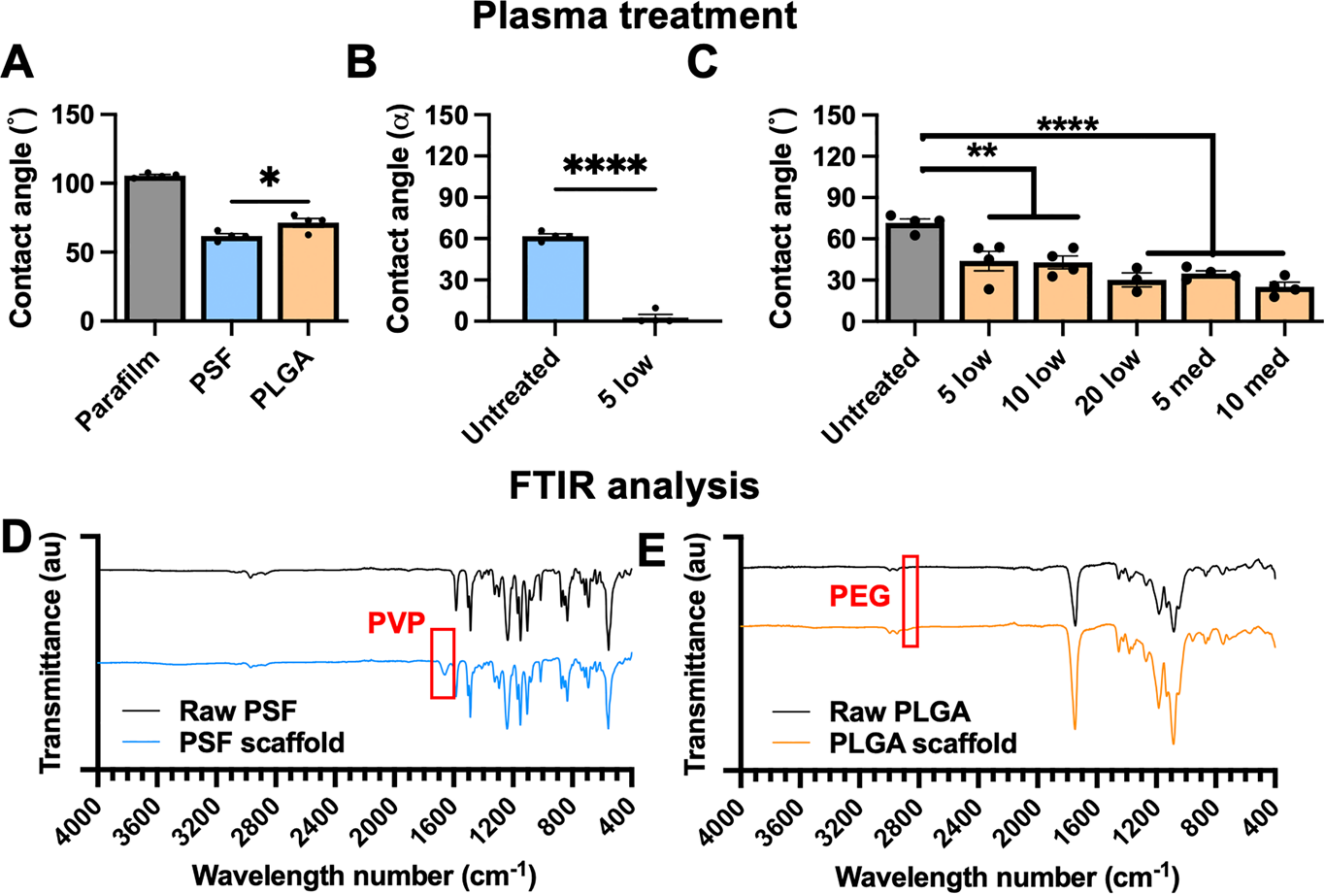
(A) Contact angle of untreated PSF and PLGA-based microtube membrane scaffolds. *N* = 4 per group, compared by unpaired *t*-test. Parafilm is shown as a hydrophobic control. (B) Contact angle of water five seconds following deposition onto treated (5 min, low power) and untreated PSF-based scaffolds. *N* = 4 per group, compared by unpaired *t*-test. (C) Contact angle of PLGA-based scaffolds after plasma treatment at different power and duration settings. *N* ≥ 3 per group, compared by ANOVA with Dunnet’s post-test. (D) FTIR analysis of bulk PSF and electrospun PSF scaffolds. The band detecting PVP porogen is highlighted with a red margin. Each plot is the average of *n* = 3 samples. (E) FTIR analysis of bulk PLGA and electrospun PLGA scaffolds. The location of a distinct PEG peak is highlighted with a red box. Each FTIR plot is the average of *n* = 3 samples.

To examine the suitability of the material for cell culture, surface contact angle was measured. The results in Figure 2A show that freshly spun, untreated PLGA scaffolds had a higher contact angle (73.1 ± 4.2°) than untreated PSF scaffolds (61.8 ± 3.3°, p = 0.032). Parafilm was more hydrophobic than either material, with a contact angle of 105.7 ± 1.7°. Immediately following plasma treatment for five minutes at low power (Figure 2B), PSF scaffolds showed a significant reduction in contact angle to 2.7 ± 4.65° (*p* = ≤ 0.001 vs untreated). PLGA scaffolds, however, showed a lesser reduction to an average of 43.9 ± 14.2° (*p* = 0.01 vs untreated) at the same settings, and so, additional conditions were analyzed, as shown in Figure 2C. Plasma treatment of PLGA samples at medium power for five minutes lowered the contact angle and produced more consistent results (34.8 ± 4.0°, *p* ≤ 0.001 vs untreated). Increasing the duration to 10 min of medium power caused the materials to display visible signs of twisting, melting, or degradation (not shown). Therefore, for future experiments, optimal settings (PSF five minutes at low power, PLGA five minutes at medium power) were used.

To determine the duration/shelf life of this improved wetting, samples were stored at room temperature and measured every 2 days for 14 days following plasma treatment. The results (Figure S1B,C) show that PSF materials lost some hydrophilicity after 2 days but the contact angle remained lower for at least 14 days. However, PLGA scaffolds rapidly lost the improved wetting, which was restored to untreated contact angles after 4 days. This is likely due to hydrophobic recovery due to oxidation reactions, which is known to occur following surface plasma treatment.^31^ Therefore, all materials were plasma treated immediately before use in future experiments.

FTIR was used to confirm the chemical makeup of the final electrospun scaffold compared to the original bulk polymer. The results plotted in Figure 2D,E display the mean of three independent analyses. One peak (1648 cm^–1^) was identified in the PSF group, which was not in the bulk polymer spectrum. This was identified as residual PVP (Figure S1D). The PLGA scaffold FTIR spectrum matched with its bulk material spectrum. For PLGA scaffolds, PEG 35k was used as porogen, which was removed by final washing steps, evidenced by the absence of a peak at 2870 cm^–1^ (Figure S1E).

#### 3.1.3 Mechanical Testing

**Figure 3:**
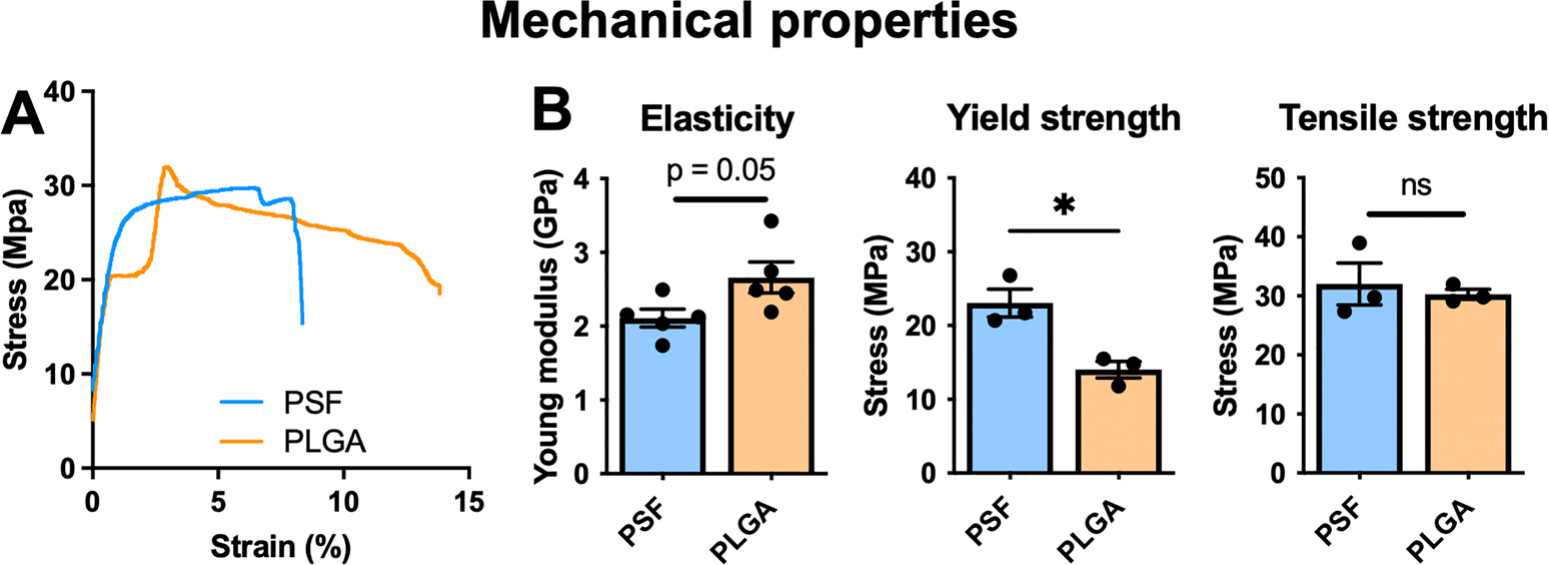
(A) Representative stress–strain curves for electrospun PSF and PLGA scaffolds. Lines show the mean of *n* ≥ 3 materials per group. (B) Calculated elasticity, yield strength, and tensile strength of electrospun PSF and PLGA scaffolds. *n* ≥ 3 scaffolds per measurement, compared by unpaired *t*-test.

For implantation into soft tissues, a flexible, elastic material would be better suited than a rigid or brittle material. In addition, pliability allows for easier handling during implantation and would reduce irritation and damage to host tissues following implantation. Therefore, some mechanical properties of both materials were tested by performing longitudinal stretching on each final fabricated material. Representative stress–strain graphs for freshly made, plasma-treated PSF and PLGA scaffolds (0.5 cm width × 4.0 cm length) are shown in Figure 3A. The linear range (*r*^2^ ≥ 0.95) of each stress–strain curve was used to calculate Youngs modulus, yield strength, and ultimate tensile strength, as shown in Figure 3B. These data are in line with previously published data showing that PSF and PLGA have an elastic modulus of approximately 2.0 GPa.^32^ However, these data only test longitudinal stretching, not bending, twisting, or other stresses. When handling, PLGA-based scaffolds were noticeably less prone to fragmenting, whereas scaffolds formed from PSF were comparatively more brittle.

Thermogravimetric analysis (TGA) was used to characterize the stability and composition of the electrospun materials. The results (Figure S2A,B) showed that both materials had high thermal stability within the range of biologically relevant temperatures. PSF scaffolds were far more thermostable overall than those formed from PLGA, as expected. Together, these results show that these scaffolds have suitable physicochemical properties for biological purposes.

### 3.2 Cell Culture

#### 3.2.1 Assessment of Cell Compatibility

**Figure 4:**
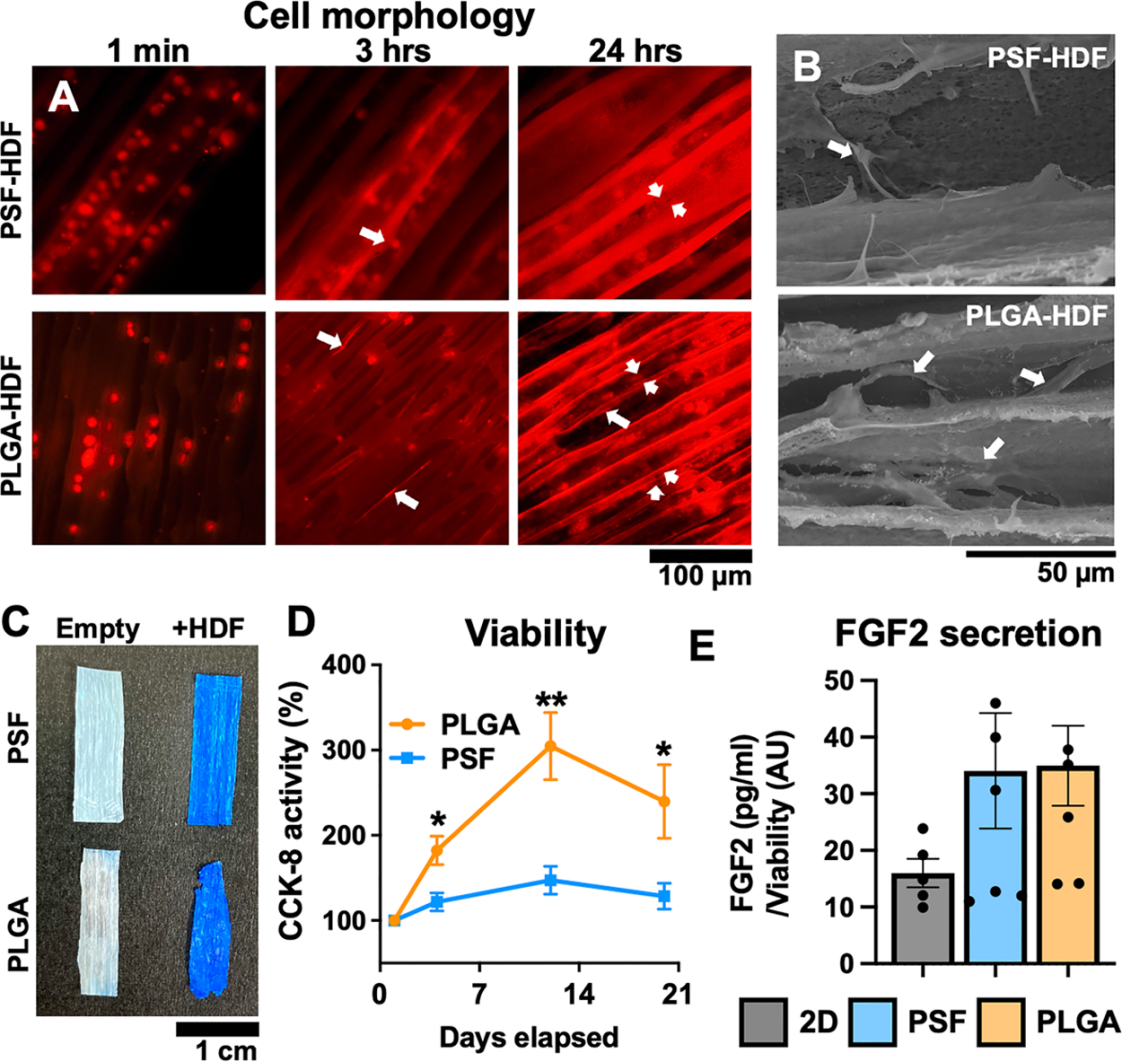
(A) Microscopy of fluorescence-labeled HDFs loaded into PSF and PLGA microtube membranes showing uptake and adhesion of cells to the inner walls of tubes. Adherent cells at three and 24 h are indicated by white arrows. Cells in distinct 3D layers at 24 h are indicated by white arrow heads. (B) SEM images showing three-dimensional cell growth (white arrows) and extracellular matrix deposition inside capillaries of PSF and PLGA-based scaffolds. The top surface of the material was removed by tape stripping. (C) Representative images of Coomassie blue-stained PSF and PLGA-based scaffolds, with (+HDF) and without cells after 3 days of culture. (D) HDF viability over time measured by CCK-8 assay following encapsulation in PSF or PLGA scaffolds. Day 1 was used as a baseline. *N* = 5 per group, except D20 PLGA (*N* = 4) due to material degradation. Time points were compared by unpaired *t*-test. (E) ELISA demonstrating normalized hFGF2 secretion following 12 days HDF encapsulation in PSF and PLGA-based scaffolds. *N* ≥ 5 samples per group.

Primary human dermal fibroblasts (HDFs) from healthy donors were chosen as a model cell to assess the material suitability for cell culture. Focus-stacked images of CellTracker-labeled HDFs (Figure 4A) show spherical cells inside the microtubes immediately following loading by capillary action. Cell attachment and flattening occurred within 180 min for PLGA-HDF and cells elongated along the capillary walls. Despite the lower contact angle measured by goniometry, some cells remained in a more spherical shape in PSF microtubes, even after 24 h. SEM of cultures after 3 days (Figure 4B) revealed spindle-shaped fibroblasts along the tube walls and abundant extracellular matrix deposition inside the microtubes, confirming 3D cell culture. After 3 days of culture, empty or cell-loaded materials were stained with Coomassie blue to visualize protein (both cells and secreted extracellular matrix) coverage. Both materials showed strong staining with complete coverage throughout the whole material, as shown in Figure 4C.

Cell viability was measured at multiple time points using a nontoxic CCK-8 assay to measure metabolic activity. The first assay was performed 24 h following cell seeding to establish a baseline, accounting for the different surface areas and volumes of each material. The assay was then repeated at day 4, day 12, and day 20. For each reading, the scaffold was moved to a fresh well with culture medium and CCK-8 reagent and incubated for four hours. The results (Figure 4D) showed that both materials successfully supported culture and preserved viability of HDFs, but the cells expanded more readily inside PLGA, reaching 3.0-fold CCK-8 activity at 12 days compared to 1.4-fold in PSF materials. Cultures were maintained for 21 days, by which time the PLGA scaffolds began to degrade and break into smaller pieces, releasing the encapsulated cells.

To confirm that cell-derived products could be successfully secreted from inside the materials, we measured secreted levels of human fibroblast growth factor 2 (hFGF2, basic-FGF) by ELISA. FGF2 is a pro-angiogenic, antifibrotic growth factor produced by many cell types, which can be used in cell therapy or as a standalone therapeutic agent.^33^ Basal culture media with empty scaffolds were used as a blank and HDFs were cultured under mild hypoxia (5% O_2_) to stimulate FGF2 release. When normalized against cell number (determined by CCK-8 assay), both PSF and PLGA-cultured HDFs produced, on average, approximately 2-fold more FGF2 than 2D-cultured cells on a “per cell” basis. This was not significantly different (*p* = 0.24 and 0.21 respectively, by ANOVA) to 2D culture; however, it confirms that cell-secreted products were successfully released from the porous microtubes into the surrounding medium.

### 3.3 Animal Implantation

#### 3.3.1 Assessment of *in Vivo* Systemic Biocompatibility

**Figure 5:**
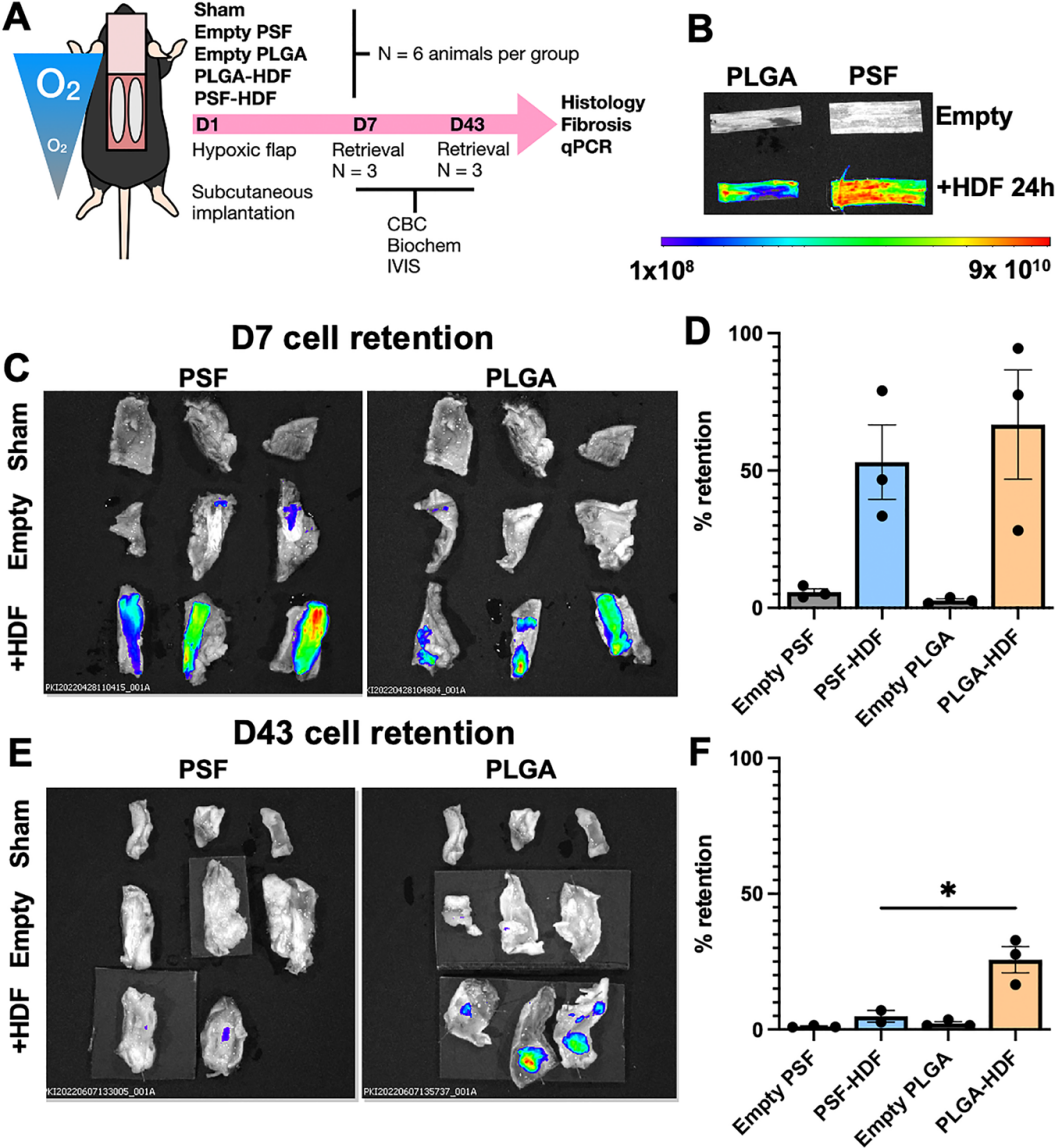
(A) Schematic diagram showing experimental design. (B) IVIS image showing empty PLGA and PSF scaffolds and those loaded with labeled HDFs. The same image scaling is used for all images shown. Units are radiant efficiency ((p/sec/cm^2^/s)/μW/cm^2^). (C) IVIS image of skin sections removed from mice after 7 days (D7). Sham-operated animals received no scaffold or labeled cells and were used as a blank. (D) Quantification of cell retention after 7 days. *N* = 3 per group (E) IVIS image of skin sections removed from mice after 43 days. Sham-operated animal samples were used as a blank. Some samples were pinned to cardboard to hold them flat for imaging. (F) Quantification of cell retention after 43 days. *N* = 3 except PSF-HDF (*N* = 2) * = *p* < 0.05, measured by ANOVA.

Next, we designed an animal experiment to assess cell retention and host response. Immunocompetent C57/BL6 mice were used, and empty or HDF-loaded scaffolds were xenotransplanted into the subcutaneous space on each flank of a hypoxic flap model, as illustrated in Figure 5A. Sham surgery was also carried out to allow for the tissue damage, inflammation, and immune response associated with the incisions and sutures. Thus, this experimental design allowed us to determine the response to the material alone and the response to the material containing donor cells. Analysis of mouse body weight (Figure S3) showed no significant difference between groups at any time point following implantation. Three mice from each group were sacrificed after 7 days, and samples of tissue/material and blood were taken for assessment of the early host response. Complete blood count (CBC) analysis (Figure S4) revealed several changes. There was no change in total erythrocyte (RBC) concentration, indicating that there was no obvious systemic hemolysis for either cell-free or cell-loaded materials. The total circulating platelet (PLT) count was decreased for both empty PSF and PSF-HDF, indicating that there may be fibrinogen deposition and platelet adhesion to the surface of the material.^34^ Total WBC count was not changed by either of the cell-free materials, but it was significantly elevated in PSF-HDF mice (2.8-fold increase, *p* = 0.006). PLGA-HDF mice also showed a 1.8-fold higher average WBC count, but this was not statistically significant (*p* = 0.244) compared to the sham group. WBC differential revealed significantly increased neutrophils (NEUT, 2.5-fold, *p* = 0.048) and lymphocytes (LYMPH, 2.8-fold, *p* = 0.006) in PSF-HDF mice. Lymphocytes were also increased in PLGA-HDF mice (2.5-fold, *p* = 0.019). Since materials without cells did not stimulate any increase in lymphocytes, this observed lymphocytosis is likely in response to the exposure of human cells to the mouse immune system, rather than the material itself. After 43 days, all altered parameters had returned to within the normal range and there were no significant differences between groups.

Biochemical analyses were also performed on serum samples from each mouse (Figure S5). At D7, there was no change in blood urea nitrogen (BUN), indicating that kidney function was normal in all groups. Alanine transaminase (ALT) was also unchanged in all groups. Serum albumin (ALB) was significantly lower in empty PSF mice, which, in combination with normal ALT, likely indicates increased capillary permeability and subsequent edema in response to inflammation.^35^ Lactate dehydrogenase (LDH), a nonspecific marker of tissue injury, was raised in both empty PSF and PSF-HDF mice but was unchanged in empty PLGA and PLGA-HDF animals. After 43 days, LDH had fallen in all animals, and there were no significant differences between groups.

#### 3.3.2 Assessment of Donor Cell Retention

Cell retention was visualized by IVIS measurement. Empty materials showed a negligible amount of background signal (<5%), whereas HDF-loaded materials showed a very strong signal, as shown in Figure 5B. Scaffolds seeded at matching time points and cultured *in vitro* were used as controls to represent 100% expected retention. Background was normalized using tissues from sham surgery animals in the same image frame. Quantification of D7 samples (Figure 5C,D) revealed that PSF-HDF showed 53.05 ± 13.54% retention and PLGA-HDF showed 66.73 ± 19.88% retention, which were not significantly different to one another. At D43 (Figure 5E,F), counter to our expectations, we measured that cell retention was significantly (*p* = 0.04) greater in PLGA-HDF mice (25.71 ± 4.82%) than PSF-HDF mice (4.87 ± 2.14%). The PSF material was still visible to the naked eye as a white rectangle embedded under fibrotic tissue but had negligible cell signal by IVIS in all mice. On the other hand, PLGA materials showed only small areas of remaining material, but those areas still showed strong cell signals.

#### 3.3.3 Tissue Morphology Following Implantation

**Figure 6:**
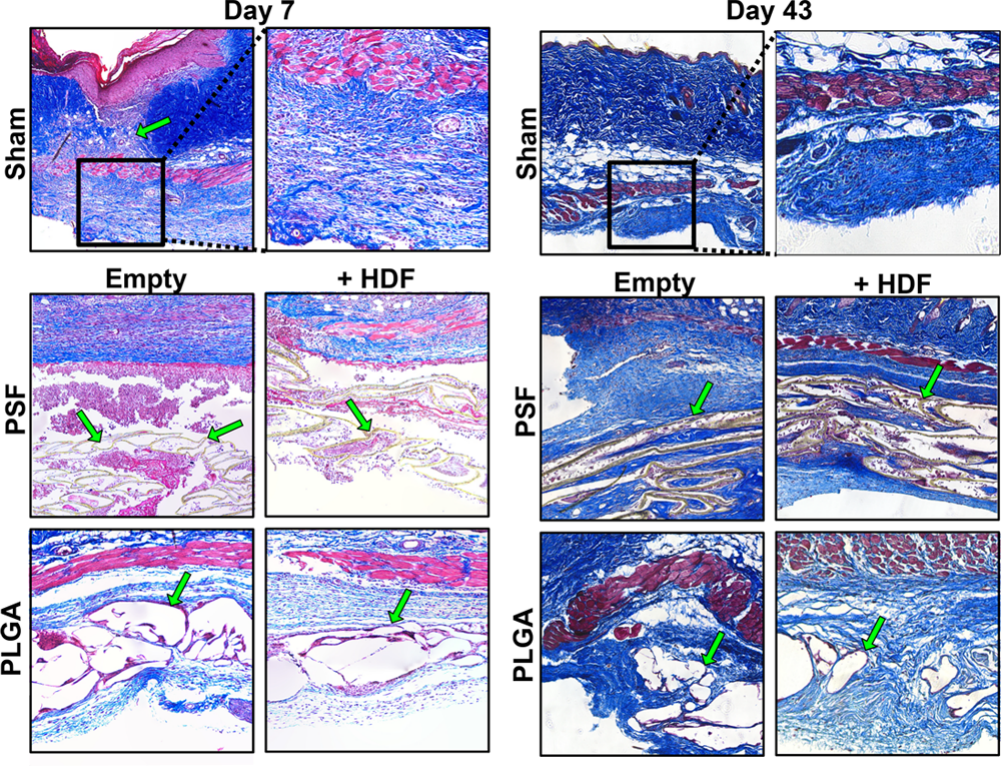
Masson’s Trichrome staining of skin sections seven and 43 days following hypoxia induction and material/cell implantation. All animals were examined, and representative images are shown. The sham group shows the location of the magnified section, underneath the wound/incision area (annotated by green arrows). In the implantation groups, the scaffold is annotated with green arrows.

To explore this further, sections were stained with Masson’s Trichrome stain, as shown in Figure 6. The images show the material implant location below the skin, annotated by green arrows. The sham group shows a clear injury region with disruption of the dermis and a thicker epidermis. The images revealed that PSF-based scaffolds were highly fragmented, with visible gaps in the microtube walls and some tubes were filled with cells. This may explain the lower-than-expected HDF retention which we detected by IVIS. On the other hand, PLGA-based materials showed distortion of the microtubes but the tube walls appeared mostly intact and the tubes did not contain obvious cell infiltration after 7 days. The PLGA materials were covered by a loose, thin layer of collagenous (blue stained) tissue, rich in spindle-shaped cells. On the other hand, PSF scaffolds were surrounded by a thick layer of erythrocytes (pink-stained anuclear cells with distinct morphology), small cell fragments (typical of platelets), and abundant red-stained protein, which may be fibrin, fibrinogen, and other proteins, which typically adhere to implants.^36^ Trichrome images from D43 show collagenous tissues surrounding both materials. A layer of adsorbed protein and many host cells were clearly seen inside and outside the PSF microtubes. Although fragmented, the PSF material was still easily visible, with a similar appearance to D7. On the other hand, small areas of remaining PLGA scaffolds were surrounded by looser fibrous tissue and fewer host cells. Areas where the PLGA scaffold had degraded appeared no different to sham-operated animals, indicating that the material had been cleared without visible adverse effects.

#### 3.3.4 Host Response

**Figure 7:**
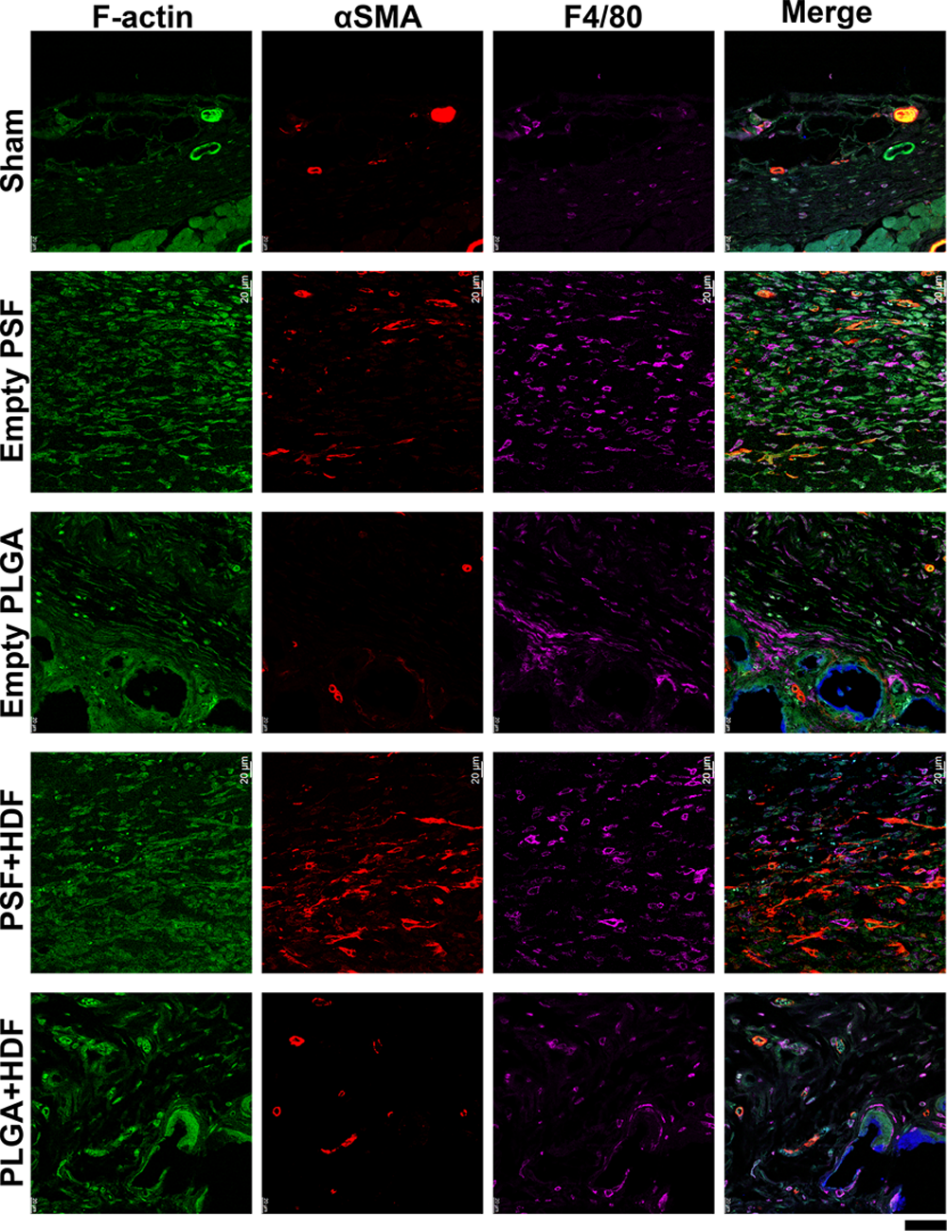
Immunofluorescence staining of immune cell markers at the material implantation site after 43 days. Blue in the merged image shows CD3 staining. Scale bar 50 μm.

Immunofluorescence staining (Figure 7) was carried out at D43 to assess the host response. This revealed that the implanted materials were surrounded by host myofibroblasts (αSMA^+^, red, spindle-shaped cells). PLGA-based materials displayed noticeably less αSMA staining than PSF scaffolds. Both materials had macrophage (F4/80, magenta) staining, with empty PSF and PSF-HDF showing more abundant macrophage tissue infiltration, while the PLGA groups showed macrophages more closely associated to the material surface. CD3 positive cells (T-cells, blue/BV421 staining) were not numerous in the sections, but more cells were noted in the PSF-HDF groups. High-magnification images of CD3 staining are shown in Figure S6.

#### 3.3.5 Assessment of Host Response by Gene Expression

**Figure 8:**
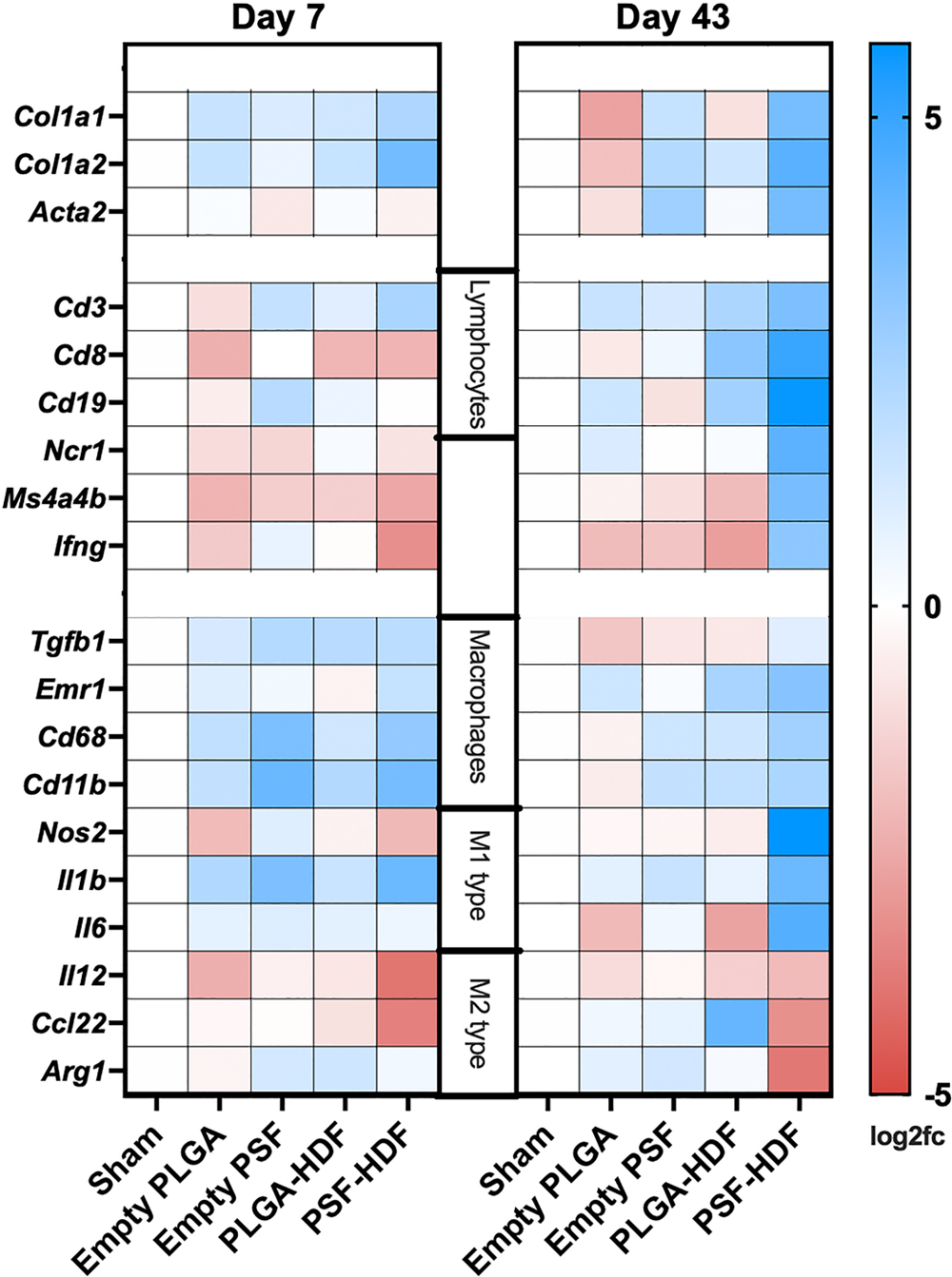
Heatmap showing changes in mouse gene expression of tissue surrounding the implant site. Changes are shown as log2 fold-change compared to sham-operated mice. *N* = 3 mice per group, except PSF-HDF D43 (*N* = 2). Genes related to host tissue remodeling, inflammation, and the immune response are shown.

To gain an overall profile of the host response, qPCR was used to measure gene expression levels of mouse immune cell markers and tissue remodeling. Samples were taken from the distal area of the skin flap, which would have had the lowest oxygen concentration. The results (Figure 8) showed only modest differences at D7. The largest differences were observed in macrophage markers *Cd68* and *Cd11b*, which were higher in mice receiving PSF-based scaffolds, and cytokines such as *Il1b* and *Tgfb1*, which were elevated in all animals that received implants compared to the sham group. At D43, the differences between groups were much more apparent. PSF-HDF mice showed elevated (log2 fc ≥3.0) markers of tissue remodeling (*Col1a1*, *Col1a2*, *Acta2*), in agreement with the Trichrome staining. Classic “M1” macrophages (*Nos2*) and associated cytokines (*Il1b* and *Il6*) and lower expression of “M2” macrophages (*Il12*, *Ccl22*, and *Arg1*) were also noted in PSF-HDF mice.^37^ Both PLGA-HDF and PSF-HDF mice showed elevated expression (compared to sham) of T lymphocyte and B lymphocyte markers including *Cd3*, *Cd8*, and *Cd19*. This demonstrates that the encapsulated human cells were exposed to the host immune system in both materials. Overall, PLGA-based materials produced a more muted immune response than PSF-based materials. Since fibroblast-derived factors can stimulate angiogenesis, we also examined angiogenesis markers at D43, as shown in Figure S7. PLGA-HDF mice showed the highest expression of *Flt1*, *Vegfa*, *Pecam1*, and *Mcam*. These were higher than empty PLGA scaffolds alone, indicating that the live HDFs were able to stimulate angiogenic signaling in the surrounding tissues.

## Discussion

Here, we present the fabrication and use of a degradable, highly porous microtube membrane array scaffold suitable for three-dimensional cell culture and compatible with implantation in a cell therapy model. Biomaterials are well-known to offer the possibility of improving donor cell retention and survival at implantation sites.^17,38,39^ The material presented in this manuscript has numerous advantages including a large surface area for three-dimensional cell growth and pores to allow for the exchange of nutrients. The material is degradable by host tissues, allowing for minimally invasive clearance in applications where this may be desirable. In some instances, it may be desirable for an implanted material/cell to be retained for a duration of therapy lasting months or years—for example, encapsulated pancreatic islets for glucose management in diabetic patients.^40,41^ However, for applications such as regenerative medicine, a shorter treatment duration is sufficient. In these instances, a degradable material is advantageous, particularly if retrieving the implant may be invasive, such as for therapy of cardiac or brain disorders.^42^ Hydrogels formed from gelatin, alginate, hyaluronic acid (HA), or decellularized tissues are perhaps the most studied materials for cell delivery applications.^23,43^ HA is particularly advantageous since it is inherently bioactive and can promote neovascularization and endogenous stem cell recruitment, even when used standalone.^44^ HA can also improve donor cell retention at injury sites, and its degradation is relatively predictable, based on molecular weight and cross-linking.^45,46^ However, suspension into a hydrogel and injection into host tissues still subjects therapeutic donor cells to significant stresses, and hydrogels offer little protection from the host immune system. On the other hand, the PLGA scaffold described in our study allows for cell transplantation with minimal disturbance and clearly offered some protection from the immune system, as shown by the prolonged cell retention and immunofluorescence images. From a therapeutic point of view, our material lacks any inherent bioactivity, and potential benefits would have to be derived from paracrine secretions of the encapsulated cells rather than integration or differentiation.

To demonstrate cell retention, our study purposely used a xenotransplantation model in an immunocompetent mouse strain. Xenotransplantation is not clinically realistic, but it allowed us to strictly characterize the retention and survival of cells due to the material under “worst case” conditions, without the possibility of donor cells surviving and proliferating inside mouse tissues. Any nonencapsulated human cells would be rapidly eliminated by the host immune system within a few days.^47^ For the same reason, we opted not to culture mouse cells, since any future therapies would certainly utilize human cells. Lastly, we chose not to use immunocompromised mice since they show altered responses to injury and perturbed wound healing.^48^ An ischemic skin flap model was used so that we could assess the host response at an injured site with relevant inflammation, increased vascular permeability, and immune activation. Although dermal fibroblasts are not a common candidate for cell therapy, they nevertheless secrete compounds which can modulate fibrosis and increase angiogenesis.^49^ Indeed, we observed increased markers of mouse angiogenesis at D43 for the PLGA-HDF group. In terms of safety and feasibility, blood tests revealed that PLGA-based scaffolds provoked a milder host response than a permanent PSF-based material. Tissue histology revealed less adhesion of proteins, erythrocytes, and platelets to PLGA than PSF scaffolds. This is not surprising, since PLGA-based materials are widely used in the clinic and are known to be well-tolerated.^25,26^ In our experimental design, immune activation, cell recruitment, and inflammation would still be expected due to the xenotransplantation model. However, in a clinical situation using autologous cells or other nonimmunogenic donor cells, this would likely be much less significant.

Counter to our original hypothesis, we found that degradable PLGA-based materials demonstrated longed cell retention than nondegradable materials. While PSF-based materials were chemically stable, the evidence in this manuscript suggests that they provoked a more hostile immune response, particularly when combined with xenogeneic cells. Reduced cell retention may also be in part due to the physical properties of the material, since the PSF scaffolds appeared to fragment early after implantation, thus exposing more human antigens to the mouse immune system.

Regarding the scaffold fabrication, electrospinning the structure using PLGA presented significant technical challenges. In our first fabrication attempts, PLGA scaffolds formed tubes that were too narrow for cell loading (not shown). To increase the tube dimensions we increased the core and/or shell solution flow rates; however, this resulted in twisting or collapsing of the walls. Next, we increased the PEG and PEO concentration to increase the core solution viscosity, but this resulted in failure to electrospin. The problem was overcome by adjusting the concentration of shell solution to promote more rapid solvent evaporation, which then allowed for large tube diameters. The material presented in this manuscript is the optimized fabrication method producing a stable morphology, suitable porosity, and cell loading.

This study is not without some limitations. First, the hollow tube structure of the material does not allow for conventional porosimetry to be used to determine surface porosity. Therefore, we relied on electron microscopy to measure surface pore diameter, which may not fully reflect the diffusion pathway of nutrients and secreted factors. Metrics such as cell viability and growth factor secretion were used to confirm successful nutrient exchange. Another limitation is that we were only able to measure cell retention in *ex vivo* samples rather than tracking cell retention in the live animals. Lastly, we were unfortunately unable to measure reperfusion of the tissue flap by laser doppler flowmetry to directly compare therapeutic efficacy of each treatment group. Instead, we used immunofluorescence staining and gene expression as proxy measurements. In the future, we aim to explore this PLGA scaffold platform further, using alternative cell types in injury models which better represent human pathologies.

## Conclusion

This manuscript presents a degradable, PLGA-based scaffold for cell culture and implantation. The material degraded completely within less than two months under *in vitro* conditions and could support culture of primary human dermal fibroblasts while allowing for secretion of paracrine factors. The empty PLGA material was tolerated well by mice following subcutaneous implantation, showing only a mild host response in terms of gene expression, blood biochemistry, and hematology parameters. A construct containing donor cells was also well-tolerated and showed evidence of pro-angiogenic signaling around the implant site. By comparison, a nondegradable implant formed from polysulfone (PSF) elevated markers of tissue damage and inflammation and greater host cell recruitment to the implant site. Interestingly, we also found that the degradable PLGA material prolonged donor cell retention compared to nondegradable PSF. In the future, the PLGA-based implant could be used as a platform to deliver and temporarily retain therapeutic donor cells at a chosen implant site. This could potentially be applied to acute injuries such as skin wounds, bone fractures, or ischemic diseases.

## Supporting Information Available

Figures of material dimensions, plasma treatment, FTIR of PEG and PVP, thermogravimetric analysis, mouse body weight after implantation, mouse haematology results after implantation, mouse serum biochemistry results after implantation, CD3 staining at Day 43 after implantation, and gene expression of angiogenesis markers at Day 43 after implantation and table of list of primers used (PDF)
