# Supplementary material for: Degradable Biocompatible Porous Microtube Scaffold for Extended Donor Cell Survival and Activity
Source: ACS Biomater Sci Eng. 2023 Jan 3;9(2):719–31. doi: 10.1021/acsbiomaterials.2c00899 (PMC9930086; doi:10.1021/acsbiomaterials.2c00899)
Supplement: Supplementary file 1 — ab2c00899_si_001.pdf [file ab2c00899_si_001.pdf]

Degradable biocompatible porous microtube scaffold for extended donor cell survival and activity

Helen Nguyễn[1], Chien-Chung Chen[1,2], Andreas Czosseck[1], Max M Chen[1], Thomashire A. George[2,3] and David J. Lundy[1,2,4*]

1. Graduate Institute of Biomedical Materials & Tissue Engineering, College of Biomedical Engineering, Taipei Medical University, 250 Wuxing Street, Taipei 110, Taiwan
2. International Ph.D. Program in Biomedical Engineering, College of Biomedical Engineering, 250 Wuxing Street, Taipei 110, Taipei Medical University, Taiwan
3. Medical Laboratory Science and Diagnostics, College of Medicine and Allied Health Sciences, Tower Hill, Freetown, University of Sierra Leone
4. Center for Cell Therapy, Taipei Medical University Hospital, 250 Wuxing Street, Taipei 110, Taiwan

* Corresponding author: dlundy@tmu.edu.tw

Supporting Information: 9 pages total. Seven figures. One table.

Figure S1 – Material dimensions, Plasma treatment. FTIR of PEG and PVP

Figure S2 – Thermogravimetric analysis

Figure S3 – Mouse body weight after implantation

Figure S4 – Mouse haematology results after implantation

Figure S5 – Mouse serum biochemistry results after implantation

Figure S6 – CD3 staining at Day 43 after implantation

Figure S7 – Gene expression of angiogenesis markers at Day 43 after implantation

Table S1 – List of primers used

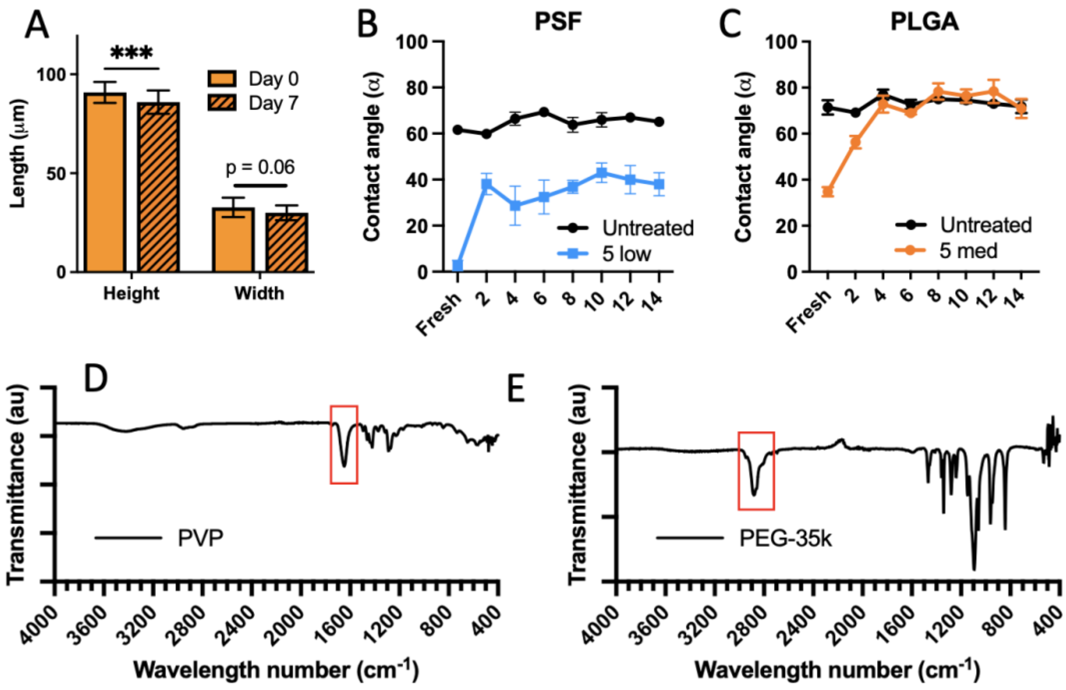

Figure S1

A. Change in PLGA scaffold tube dimensions after seven days immersion in PBS. N = 3 scaffolds per condition were analyzed, with N ≥ 20 tubes per scaffold measured. Compared by two-ANOVA with Tukey's post test. *** = P ≤ 0.001
B. Timeline of contact angle (water, 5 seconds) of plasma-treated PSF scaffolds stored under ambient conditions for 14 days. N = 4 samples per group.
C. Timeline of contact angle (water, 5 seconds) of plasma-treated PLGA scaffolds stored under ambient conditions for 14 days. N = 4 samples per group.
D. FTIR spectrum of PVP, utilised as a porogen for PSF-based materials. The signature peak at 1648 cm^{-1} is highlighted.
E. FTIR spectrum of PEG-35k, utilised as a porogen for PLGA-based materials. The characteristic peak at 2870 cm^{-1} is highlighted.

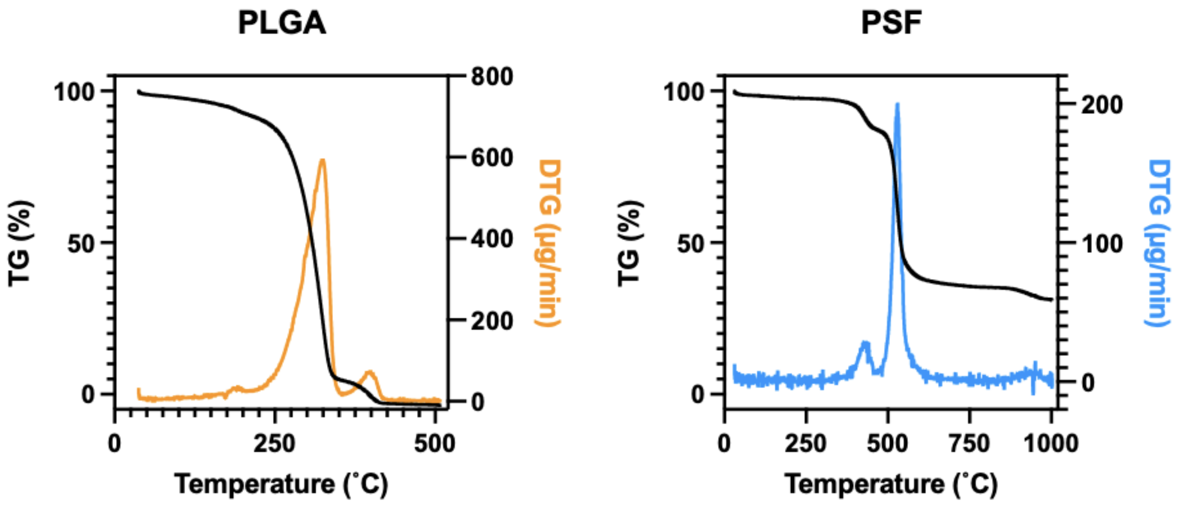

Figure S2

Thermogravimetric analysis of PLGA and PSF scaffolds

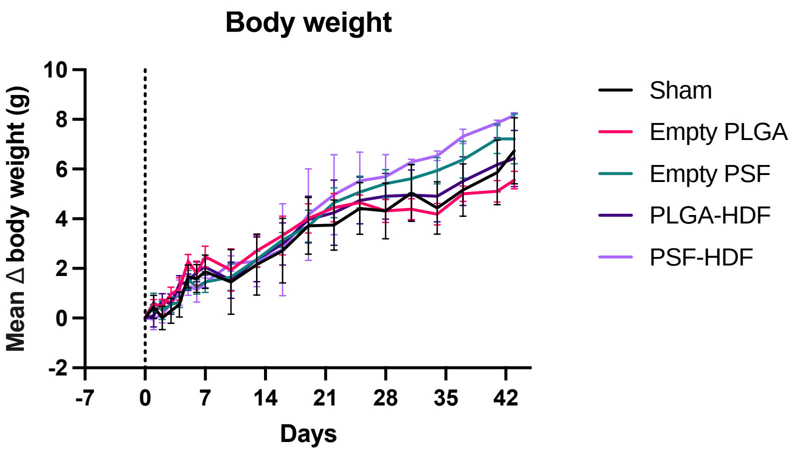

Figure S3

Changes in mouse body weight from pre-surgery to D43. N = 6 mice per group up to D7, then N = 3 mice per group up to D43, except PSF-HDF (N=2). No statistically significant differences between groups were observed.

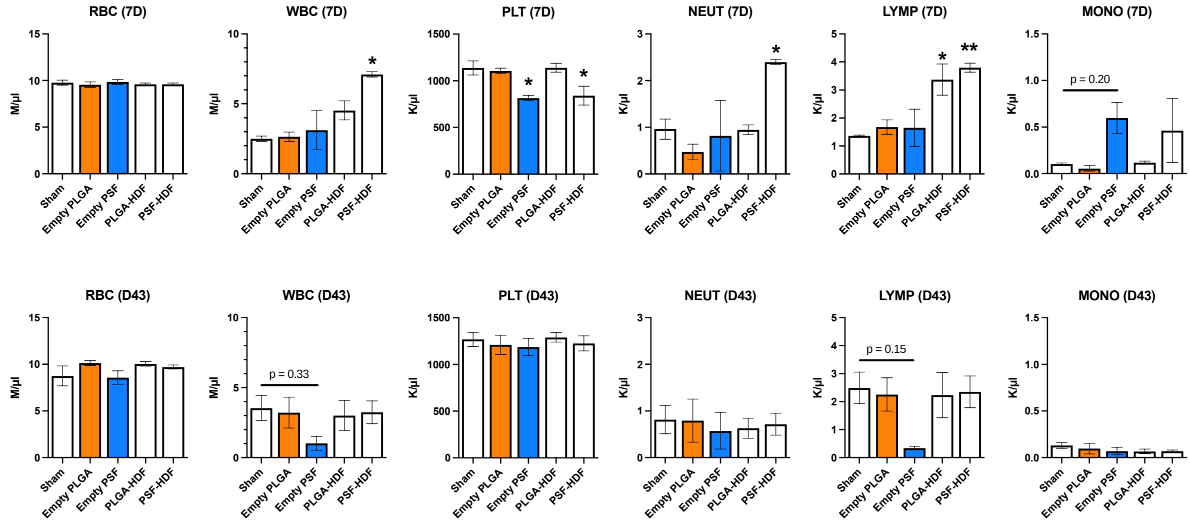

Figure S4

Complete blood count (CBC) analysis of mice at D7 and D43 following sham surgery, or implantation of empty PLGA, empty PSF, or human dermal fibroblast (HDF)-loaded PSF or PLGA. RBC = red blood cells (erythrocytes), WBC = total white blood cells, PLT = platelet count, NEUT = neutrophil count, LYMP = lymphocyte count, MONO = monocyte count. Each group was compared against sham surgery by ANOVA with Dunnett's multiple comparison test. N = 3 for all samples except PSF-HDF (N = 2) * = $p < 0.05$, ** = $p < 0.01$.

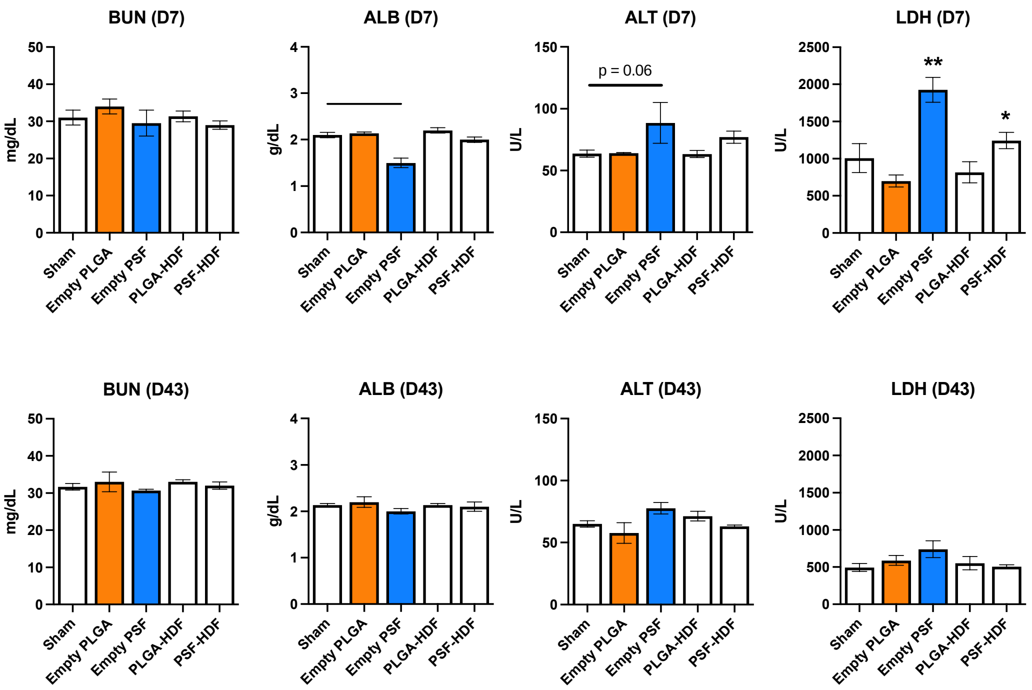

Figure S5

Serum biochemistry analysis of mice at D7 and D43 following sham surgery, empty PLGA, empty PSF, or human dermal fibroblast (HDF)-loaded PSF or PLGA microtube membrane scaffolds. BUN = blood urea nitrogen, ALB = albumin, ALT = alanine aminotransferase, LDH = lactate dehydrogenase. Each group was compared against sham surgery by ANOVA with Dunnett's multiple comparison test. N = 3 for all samples except PSF-HDF (N = 2) * = $p < 0.05$, ** = $p < 0.01$, ns = not significant.

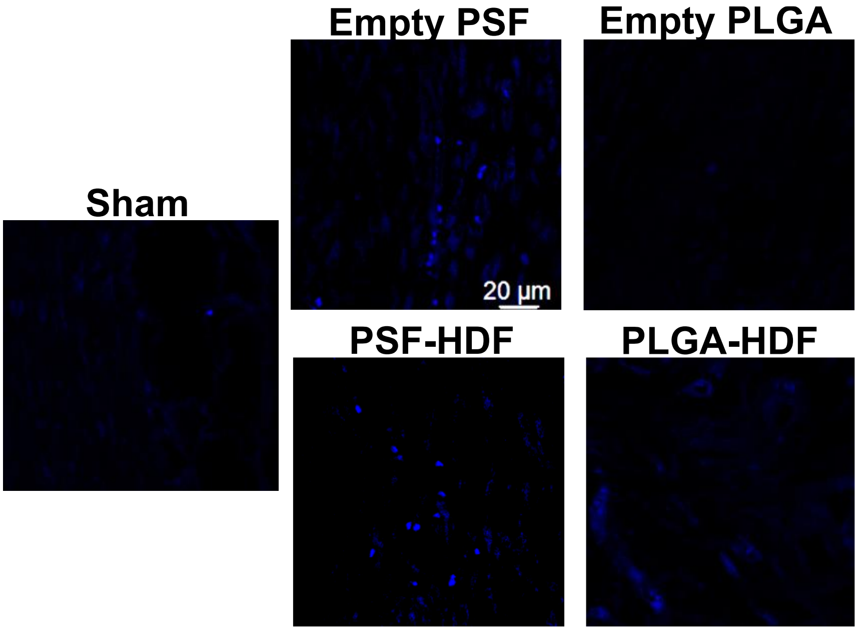

Figure S6

T-cells, labelled with anti-CD3-BV421, stained skin sections at D43 following sham surgery or implantation of empty PSF, empty PLGA or HDF-loaded PSF or PLGA microtube membrane array scaffolds.

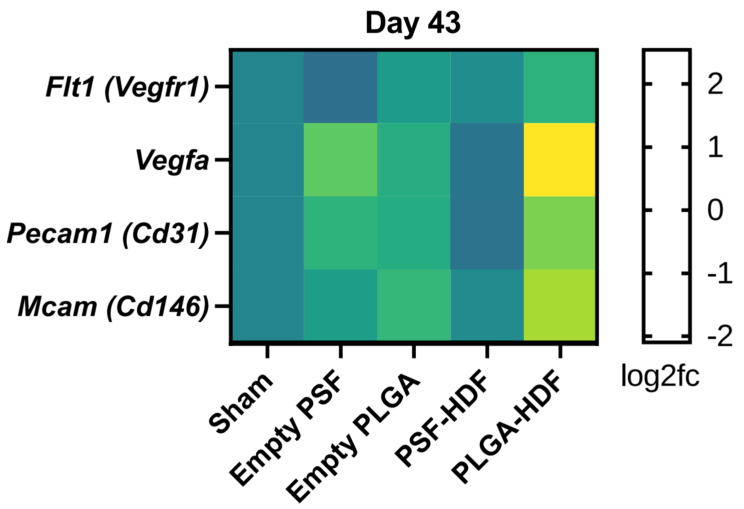

Figure S7

Gene expression of angiogenesis markers 43 days following empty material and/or material with encapsulated cell implantation. The heatmap shows log2-fold change compared to sham surgery animals. N = 3 per sample except PSF-HDF (N = 2).

Gene	Forward	Reverse	NTC CT	Sham CT
Col1a1	CATGTTCAGCTTTGTGGACCT	GCAGCTGACTTCAGGGATGT	> 40	22
Col1a2	GCAGGTTCACCTACTCTGTCC	CTTGCCCCATTCATTTGTCT	> 40	21
Acta2	CGCTTCCGCTGCCCAGAGACT	TATAGGTGGTTTCGTGGATGCCCGCT	> 39	25
Cd3	GCTCCAGGATTTCTCGGAAGTC	ATGGCTACTGCTGTCAGGTCCA	> 40	33
Cd8a	ACTACCAAGCCAGTGCTGCGAA	ATCACAGGCGAAGTCCAATCCG	> 39	34
Cd19	GCCACAGCTTTAGATGAAGGCAC	CATCCACCAGTTCTCAACAGCC	> 40	33
Ncr1	TAGGGCTCACAGAGGGACATAC	GTAGGTGCAAGGCTGCTGTTCT	> 40	32
MS4a4B	TGGGTGTAGTCAGTGTGGCTGT	AGACGGACACAGCAAGGCAGAA	> 38	29
Ifng	CAGCAACAGCAAGGCGAAAAAGG	TTTCCGCTTCCTGAGGCTGGAT	> 40	34
Tgfb1	CCTGAGTGGCTGTCTTTTGAC	ACAAGAGCAGTGAGCGCTGAAT	> 40	29
Emr1 (F4/80)	CGTGTTGTTGGTGGCACTGTGA	CCACATCAGTGTTCCAGGAGAC	> 40	28
Cd68	GCCCGAGTACAGTCTACCTGG	AGAGATGAATTCTGCGCCAT	> 38	24
Cd11b	CCAAGAGAATGCAAAAGGCTTT	GGGGGGCTGCAACAACCACA	> 38	28
Nos2	GAGACAGGGAAGTCTGAAGCAC	CCAGCAGTAGTTGCTCCTCTTC	> 37	28
Il1b	TGGACCTTCCAGGATGAGGACA	GTTCATCTCGGAGCCTGTAGTG	> 40	28
Il6	TACCACTTCACAAGTCGGAGGC	CTGCAAGTGCATCATCGTTGTTC	> 40	27
Il12	ACGAGAGTTGCCTGGCTACTAG	CCTCATAGATGCTACCAAGGCAC	> 38	31
Ccl22	GTGGAAGACAGTATCTGCTGCC	AGGCTTGCGGCAGGATTTTGAG	> 39	30
Arg1	CATTGGCTTGCGAGACGTAGAC	GCTGAAGGTCTCTTCCATCACC	> 40	29
Flt1 (Vegfr1)	TGGATGAGCAGTGTGAACGGCT	GCCAAATGCAGAGGCTTGAACG	> 38	32
Pecam1 (Cd31)	CCAAAGCCAGTAGCATCATGGTC	GGATGGTGAAGTTGGCTACAGG	> 35	26
Vegfa	CTGCTGTAACGATGAAGCCCTG	GCTGTAGGAAGCTCATCTCTCC	> 37	24
Mcam (Cd146)	CGAGGCAGAAAGTAACCAGGAC	GTCTCACGTTGTTTAGCTGGAGG	> 40	25
Gapdh	CATCACTGCCACCCAGAAGACTG	ATGCCAGTGAGCTTCCCGTTCAG	> 40	20

Table S1

List of primers used for reverse transcriptase semi-quantitative PCR. Forward and reverse sequences are shown. The cycle threshold (CT) values for non-template control (no cDNA) and typical CT range for D43 sham surgery samples is shown.
